# Cannabidiol-Loaded Lipid-Stabilized Nanoparticles Alleviate Psoriasis Severity in Mice: A New Approach for Improved Topical Drug Delivery

**DOI:** 10.3390/molecules28196907

**Published:** 2023-10-02

**Authors:** Mark Zamansky, Doron Yariv, Valeria Feinshtein, Shimon Ben-Shabat, Amnon C. Sintov

**Affiliations:** 1 Department of Biomedical Engineering, Ben Gurion University of the Negev, Be’er Sheva 84105, Israel; markzam@post.bgu.ac.il; 2 Laboratory for Biopharmaceutics, E.D. Bergmann Campus, Ben-Gurion University of the Negev, Be’er Sheva 84105, Israel; yarivdo@post.bgu.ac.il; 3 Department of Biochemistry and Pharmacology, Ben Gurion University of the Negev, Be’er Sheva 84105, Israel; shteiman@bgu.ac.il

**Keywords:** cannabidiol-loaded nanoparticles, lipid-stabilized nanoparticles, skin permeability, IMQ induced psoriasis, interleukin release

## Abstract

Cannabidiol (CBD) is a promising natural agent for treating psoriasis. CBD activity is attributed to inhibition of NF-kB, IL-1β, IL-6, and IL-17A. The present study evaluated the anti-psoriatic effect of cannabidiol in lipid-stabilized nanoparticles (LSNs) using an imiquimod (IMQ)-induced psoriasis model in mice. CBD-loaded LSNs were stabilized with three types of lipids, Cetyl alcohol (CA), Lauric acid (LA), and stearic-lauric acids (SALA), and were examined in-vitro using rat skin and in-vivo using the IMQ-model. LSNs loaded with coumarin-6 showed a localized penetration depth of about 100 µm into rat skin. The LSNs were assessed by the IMQ model accompanied by visual (psoriasis area severity index; PASI), histological, and pro-psoriatic IL-17A evaluations. Groups treated with CBD-loaded LSNs were compared to groups treated with CBD-containing emulsion, unloaded LSNs, and clobetasol propionate, and to an untreated group. CBD-loaded LSNs significantly reduced PASI scoring compared to the CBD emulsion, the unloaded LSNs, and the untreated group (negative controls). In addition, SALA- and CA-containing nanoparticles significantly inhibited IL-17A release, showing a differential response: SALA > CA > LA. The data confirms the effectiveness of CBD in psoriasis therapy and underscores LSNs as a promising platform for delivering CBD to the skin.

## 1. Introduction

**Figure 1:**
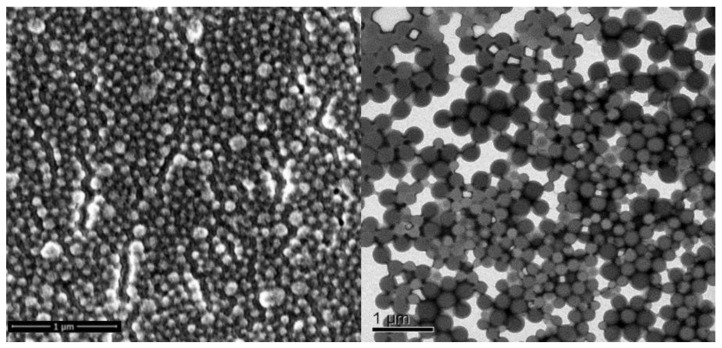
A representative scanning electron micrograph (SEM) (**left**) and transmission electron micrograph (TEM) (**right**) of cetyl alcohol (CA)-stabilized LSNs.

There has been growing evidence related to the anti-inflammatory activity of cannabidiol (CBD). CBD, one of the main components of the *Cannabis sativa* extract, is a non-psychoactive phytocannabinoid shown to have therapeutic potential for various disease states [1]. Whereas CBD acts as an antagonist or partial agonist via allosteric binding to CB1 and CB2 receptors [2,3,4], its anti-inflammatory activity is attributed to its effect exerted on the adenosine A_2_A receptor [5]. This mechanism was demonstrated in LPS-induced inflammation in a mouse model, where CBD reduced the production of pro-inflammatory cytokines (TNF-α and IL-6) and chemokines (MCP-1 and MIP-2). A study on Toll-like receptor (TLR)-activated human monocytes showed that CBD modulated the production of TNF-α, IL-1β, and IL-6 [6]. In addition, CBD is effective in treating psoriasis [7,8]. Such activity of CBD is explained by its ability to inhibit TNF-α-induced NF-kB transcription in a dose-dependent manner in HaCaT cells [9]. The potential applicability of CBD to psoriasis treatment is well established, however, its cutaneous delivery and retention in the skin remain to be addressed. The retention and prolonged mode of action are significant in chronic dermatological conditions such as psoriasis due to increasing efficacy, patient compliance, and chances of successful treatment. Formulation of nanoparticles (NPs) is a technology platform that enables active substances to penetrate the skin, retain them in the skin, and control the release of these active compounds in skin layers. The mechanisms of skin permeation and retention of nanoparticles include an entry of the applied NPs through the hair follicles [10,11,12] and penetration of the nanoparticles through the stratum corneum between corneocytes [13,14]. Published reports on the topical application of CBD in a nanoparticulate system are relatively limited. Lodzki et al. reported successful transdermal delivery of CBD using an ethosome-based formulation with 3% *w*/*w* CBD and 40% *w*/*w* ethanol combined with a phospholipid carbomer gel [15]. Another study reports successful trans-corneal delivery of CBD loaded in mixed polymeric micelles of chitosan/polyvinyl alcohol and polymethyl methacrylate under air-liquid and liquid-liquid conditions [16]. In recent publications by our group, we have shown that ethyl cellulose NPs stabilized by various lipids (Figure 1) could deliver CBD into and through rat skin [17]. We also showed that the stabilizing lipids affected the in vitro release of CBD and its ex-vivo permeation through rat skin. Particularly, the incorporation of the relatively high melting point (m.p. 69.5 °C) of stearic acid (SA) reduced the permeation, whereas the incorporation of a eutectic mixture of lauric acid (LA) (m.p. 46.4 °C) and SA (SALA) (m.p. ~36 °C) increased skin permeation. The lipid-stabilized NPs (LSNs) showed a significant anti-inflammatory activity by reduction in IL-6 and IL-8 release in TNF-α induced HaCaT cells [18].

The aim of the current research was an in-vivo efficacy evaluation of these CBD-loaded LSNs as a new potential platform for the treatment of psoriasis in humans. This evaluation was performed using the imiquimod (IMQ)-induced psoriasis model in mice. We evaluated LSNs stabilized with different lipids in terms of their effectiveness in reducing psoriasis manifestations measured by psoriasis area and severity index (PASI) score and histological and cytokine profiles. IMQ-induced psoriasis in mice is a widely used model for assessing potential psoriasis treatments with a good translation to humans [19] when using the C57BL/6 mice strain. IMQ is a Toll-like receptor (TLR7/8) agonist that can be applied to mouse skin to elicit erythema, scaling, and keratinocyte proliferation. At the same time, the phenotype involves the induction of the IL-17/IL-23 axis cytokines [20]. The ability of CBD to reduce the production of pro-inflammatory cytokines through the A_2_A and the TLR pathways, suggests the applicability of the IMQ-induced psoriasis model for its evaluation.

## 2. Results and Discussion

### 2.1. Formulation Development

The various LSNs were first characterized for particle size, particle concentration, loaded CBD concentration, and Entrapment Efficiency (EE) (Table 1). Prior to the preclinical studies, CBD-loaded LSNs were compounded into a conveniently used topical dosage form. Various delivery forms have been previously used for nanoparticles, including directly applying the NPs dispersed in water, saline, or PBS [21] and semi-solid formulations. There are several examples of NPs formulated in an acrylate gel based on Carbopol^®^ ETD 2020 [14] or Carbopol^®^ 934 [22], hydroxypropyl methylcellulose (HPMC) [23,24], Xanthan gum [25], Poloxamer 407 [26], and colloidal silica [27]. For the LSN systems, the use of polysaccharide thickeners such as xanthan gum or modified celluloses (e.g., hydroxypropyl cellulose, hydroxyethyl cellulose, or carboxymethyl cellulose) were not utilized since these linear fiber-based polymers may hinder permeation of the nanoparticles. Thus, inorganic thickeners such as colloidal silica gel and magnesium aluminum silicates (MAS) were used. In addition, Poloxamer 407 was also considered as a candidate thickener due to its thermogelling properties.

### 2.2. In Vitro Skin Permeability of Semi-Solid LSN Formulation Development

The skin permeability of CBD from the LSN semi-solid formulations was analyzed using fresh rat skin mounted on a Franz diffusion cell system. No significant differences were observed between the skin permeation and retention of the various LSNs. However, since SALA-stabilized nanoparticles showed a relatively high CBD release in our previous study [18], they were used in skin penetration experiments as a representative LSN system. The LSN dispersion was thickened with fumed silica (CAB-O-SIL^®^ 530), MAS (Veegum HV), and Poloxamer 407 thermogel, while LSNs dispersed in water served as a non-gelled control. Volumes of 0.2 mL (about 0.2 g) from the various LSN formulations, each containing CBD at a concentration of 500 µg/g, were applied on the excised skin, providing 100 µg dose of CBD over 1.77 cm^2^ of skin surface area. (i.e., 100 µg CBD/0.2 g quantity and 56.5 µg/cm^2^ skin surface area). The unstirred volume of these LSN products (about 0.2 mL) was relatively high for typical dermal application, and only a tiny portion of CBD in the SLNs came in direct contact with the skin. Typically, semi-solid formulations are applied to the skin in much lower volumes per cm^2^, constituting about 1 µL/cm^2^ [28,29] and accompanied by rubbing. Thus, considerably higher availability of LSNs would be expected in clinical use. Considering the hindrance caused by the gel structures, the total permeation from the liquid aqueous dispersion was expected to be the highest.

**Figure 2:**
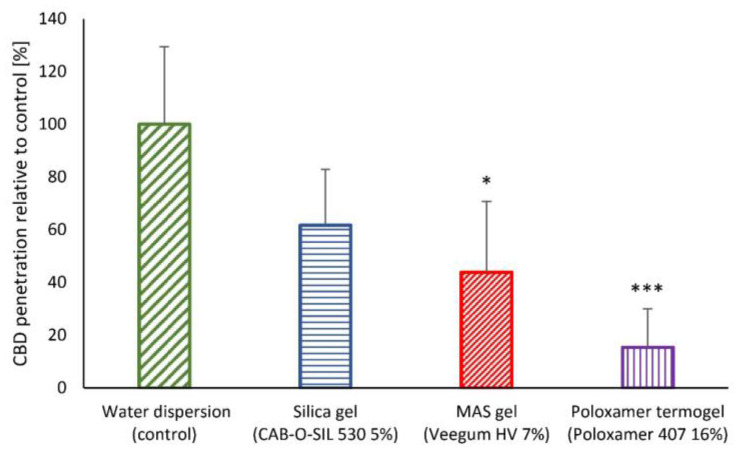
CBD penetration following the application of NP formulated with different gelling agents recovered from the skin. The values are presented relative to NP dispersion in water (100%), * *p* < 0.05, *** *p* < 0.001 ANOVA followed by Tuckey post-hoc analysis. Data are presented as mean ± SD.

As seen in Figure 2, there was a quantitative penetration of CBD into rat skin. The extent of CBD penetration depended on the type of gelling agent used for formulating the LSNs. Skin penetration of CBD from the liquid dispersion of the LSNs was relatively high, as expected (100% ± 28%, with actual values of 2.0 ± 0.8 µg/cm^2^), compared to the penetration of CBD from the poloxamer thermogel, which was the lowest (15.4% ± 14.6%, with actual values of 0.4 ± 0.3 μg/cm^2^; *p* < 0.001, ANOVA test). The relatively low skin penetration of LSNs from the poloxamer gel can be explained by the micelle density formed by poloxamers. Liu et al. [30] found that the micellar face-centered cubic lattice length is 29.5 nm for 15–45% *w*/*w* poloxamer 407 in an aqueous solution. Thus, with such small distances between the poloxamer micelles, the mobility of the 200 nm diameter LSNs through the Poloxamer gel network should have been significantly obstructed at rest (without external mechanical shear, i.e., rubbing). MAS particles are extremely thin, negatively charged plates approximately 2 nm thick and about 200–500 nm long, forming a three-dimensional network after water dispersion [31]. CAB-O-SIL^®^ 530 fumed silica has an average aggregate size of 200–300 nm, though the distance between the aggregates may be even greater. According to localization and hopping theory [32,33], the permeation of nanoparticles through an entangled polymer network depends on the confinement ‘parameter C’ described as the ratio of effective particle diameter to the effective tube diameter, which roughly corresponds to the distance between the polymer or mesh crosslinks. Particles with the confinement parameter C < 1 permeate relatively free, whereas the movement in the mesh of particles with 1 < C < 3 is due to hopping, given a sufficient activation energy. According to this theory, the diffusion coefficient in the mesh quickly drops proportionally to the exponent of (-C^2^). Thus, CAB-O-SIL^®^ 530 gel with the largest distances between the gel crosslinks would have the highest diffusion coefficient compared to the other tested gels, explaining the differences observed in Figure 2. It was also noted that the skin penetration of CBD was dependent on the silica concentration. Compared to 5% *w*/*w* gel, penetration extent decreased to 63.5 ± 17.5% for 4% *w*/*w* gel and further to 33.5% ± 7.4% for 3% *w*/*w* gel. This result is counter-intuitive to the expectation that reducing the gelling agent’s content would increase the molecules’ or NPs’ permeation through its polymeric network. It has been shown by Binder et al. [34] that an increase in the concentration of hydroxypropyl methylcellulose (HPMC) and hydroxyethyl cellulose (HEC) reduced the permeation of sulphadiazine sodium through the skin, which was explained by increased viscosity and entanglement of these cellulosic gels. In contrast to these polymers, the higher concentration of CAB-O-SIL^®^ 530 silica gel increased skin permeation, probably due to the hydrophobicity of the silica particles. Additional silica particles tend to form aggregates, building a mesh with thicker strands-fibers and larger openings. These results established CAB-O-SIL^®^ 530 5% gel as a more suitable semi-solid vehicle for LSNs and NPs in general.

### 2.3. In-Vivo Skin Penetration and Retention

**Figure 3:**
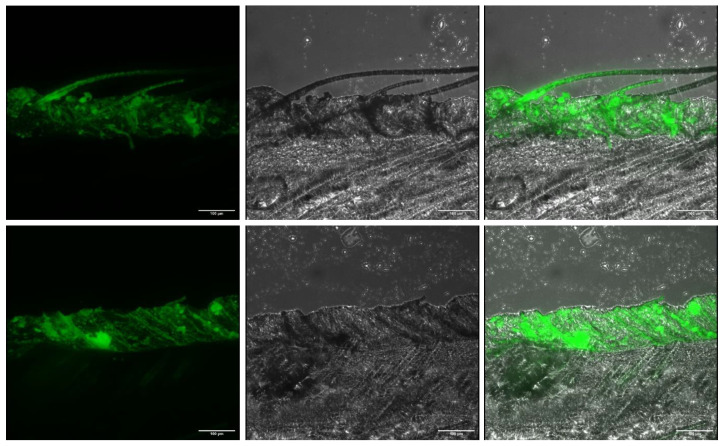
Microphotographs of excised rat skin after application of 5% CAB-O-SIL^®^ 530 gel with coumarin-6 loaded LSNs on rat skin, unshaved (**top**) and shaved (**bottom**). Left: green, fluorescent light; Middle: bright field; Right: merged pictures. Scale bar: 100 µm.

Further assessment of the skin penetrability of LSNs from the topical silica gel vehicle was performed in an in-vivo study using SALA-stabilized NPs loaded with a fluorescent marker—coumarin 6 (C6) [35]. The LSN dispersion was combined with CAB-O-SIL^®^ 530 to obtain a 5% silica gel with 7.5 × 10^11^ NPs/g. Then, the gel was applied for 2 h onto the abdomen of an anesthetized rat, which had previously been shaved and depilated as described in the experimental section. As seen in Figure 3, C6 penetrated to about 100 µm skin depth, roughly corresponding to or slightly exceeding the thickness of the epidermis layer of rat skin [36,37].

### 2.4. Imiquimod-Induced Psoriasis Model

IMQ-induced psoriasis in mice is a widely accepted psoriasis-like model for testing potential treatments with a good translation power to humans [38], especially when performed on the commonly used C57BL/6 mice strain [19]. IMQ-induced psoriasis study conducted on Cannabinoid 2 Receptor (CB2R) knockout mice showed that CB2R deficiency exacerbated psoriasis disease [39]. These results further suggest the IMQ model’s applicability for the evaluation of CBD as a treatment for psoriasis. This report presents for the first time an evaluation of CBD in IMQ-induced psoriasis model in mice, particularly the evaluation of CBD delivered by LSNs. We have previously shown that incorporating different stabilizing lipids in ethyl cellulose NPs could influence the rate and extent of both release and dermal permeability of the loaded CBD [18]. By using the IMQ-induced psoriasis model, we compared the effectiveness of three types of LSNs stabilized with either CA, LA, or SALA to the efficacy of emulsified CBD solution (‘free CBD’ or f-CBD), clobetasol (CLO, positive control), and unloaded nanoparticles (Unloaded NP, negative control).

#### 2.4.1. Erythema and Scaling

**Figure 4:**
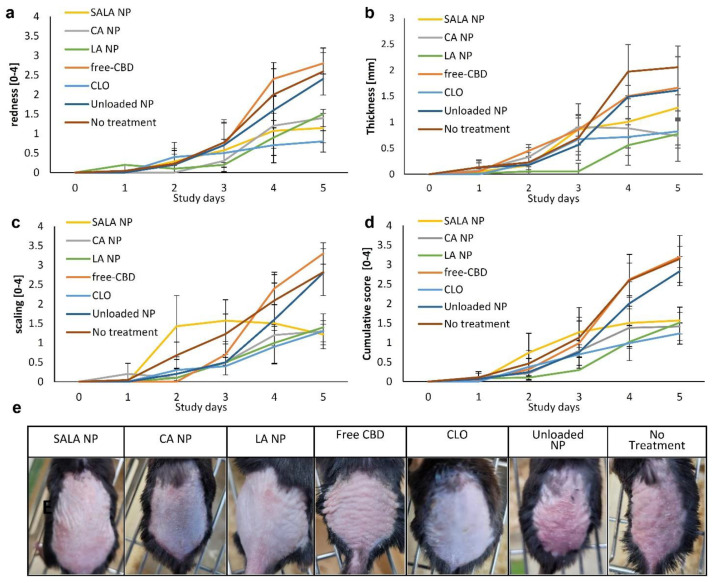
Evaluation of dorsal skin from Day 0 (D0) to Day 5 (D5) according to PASI score during IMQ induction of psoriasis-like dermatitis. Observed criteria are skin redness (**a**), thickness (**b**), scaling (**c**), and total cumulative PASI score (**d**). Representative images of mice in each group on Day 5 (D5) (**e**). Data are presented as mean ± SD.

The first appearance of IMQ-related symptoms was on the second day after induction. If not treated, the symptoms increased continuously during the following days. As shown in Figure 4d (cumulative PASI score), a significant improvement was observed in the treatment groups, CBD-loaded CA-NPs, LA-NPs, and SALA-NPs, as well as CLO (positive treatment groups—G+), compared to the apparent progression of the disease in the non-treatment (NT) group, the treatment with unloaded LSNs group, and the treatment with CBD solution (f-CBD) group (negative treatment groups—G−). On study day 4, the NT and treatment with f-CBD were significantly less effective than all G+ treatments (*p* < 0.001 for CBD-loaded LA-NPs and CLO, and *p* < 0.01 for CBD-loaded CA-NPs and CBD-loaded SALA-NPs, ANOVA). In addition, treatment with CBD-unloaded LSNs had lower effectiveness than treatments with CBD-loaded LA-NPs and CLO (*p* < 0.05, ANOVA). On the last day of the study (day 5), the differences between each treatment of G+ groups and each treatment of G- groups were highly statistically significant (*p* < 0.001, ANOVA). Similarly, no significant differences in erythema symptoms were observed between any of the treatments during the first three days. Only on day 4, a significant difference was observed between the f-CBD treatment group and each of the treatments in G+ groups (*p* < 0.001 for CLO and CBD-loaded LA-NPs, *p* < 0.01 for CBD-loaded CA-NPs and CBD-loaded SALA-NPs, Figure 4a). On day 5, all treatments in the positive G+ treatment groups were significantly more effective in the prevention of erythema than each of the treatments in the negative groups (*p* < 0.001 for NT and f-CBD groups compared to CBD-loaded CA-NPs, CBD-loaded LA-NPs, CBD-loaded SALA-NPs, and CLO; *p* < 0.001 for treatment with unloaded-LSNs compared to CBD-loaded SALA-NPs and CLO, *p* < 0.01 for treatment with unloaded-LSNs compared to CBD-loaded CA-NPs, and *p* < 0.05 for treatment with unloaded-LSNs compared to CBD-loaded LA-NP, ANOVA). As seen in Figure 4c, skin scaling was significantly minimized by CBD-loaded CA-NPs and CBD-loaded LA-NPs compared to the untreated group. In contrast, scaling increased on treatment with CBD-loaded SALA-NPs on days 2 and 3, but on day 4, these nanoparticles hindered the scaling progress. Thus, on day 4 the NT group and the f-CBD group were significantly less effective than any treatment in the positive G+ groups (*p* < 0.001 for CLO and CBD-loaded LA-NPs; *p* < 0.01 for CBD-loaded CA-NPs and CBD-loaded SALA-NPs, ANOVA). Unloaded LSNs were less effective than treatments with CLO and CBD-loaded LA-NPs (*p* < 0.05, ANOVA). On day 5, all treatments in the G+ groups were significantly more effective than those in the negative G- groups (*p* < 0.001, ANOVA).

#### 2.4.2. Skin Thickness

A gradual increase in skin thickness, another parameter of inflamed area, was observed in the NT group and the unloaded LSNs treatment group (Figure 4b). However, significant inhibition was noted by CBD-loaded LA-NPs and CBD-loaded CA-NPs (days 4 and 5). Clobetasol cream also inhibited skin thickening on days 4 and 5. All treatments in the G+ groups were significantly more effective on day 4 than the NT group (*p* < 0.001, ANOVA). Treatments with f-CBD and unloaded LSNs were significantly less effective in reducing skin thickening when compared to treatment with CBD-loaded LA-NPs (*p* < 0.001, ANOVA). On day 5, NT group showed a significant skin thickening compared to each treatment in G+ (*p* < 0.001 for CA-NP, LA-NP, and CLO and *p* < 0.01 for SALA-NP, ANOVA), f-CBD treatment was also less effective compared to treatments with CBD-loaded CA-NPs, CBD-loaded LA-NPs, and CLO (*p* < 0.05, ANOVA). At the same time, unloaded LSNs were significantly less effective than treatments with CBD-loaded CA-NPs and CBD-loaded LA-NPs only (*p* < 0.05, ANOVA).

#### 2.4.3. Cumulative Day-to-Day Scoring

**Figure 5:**
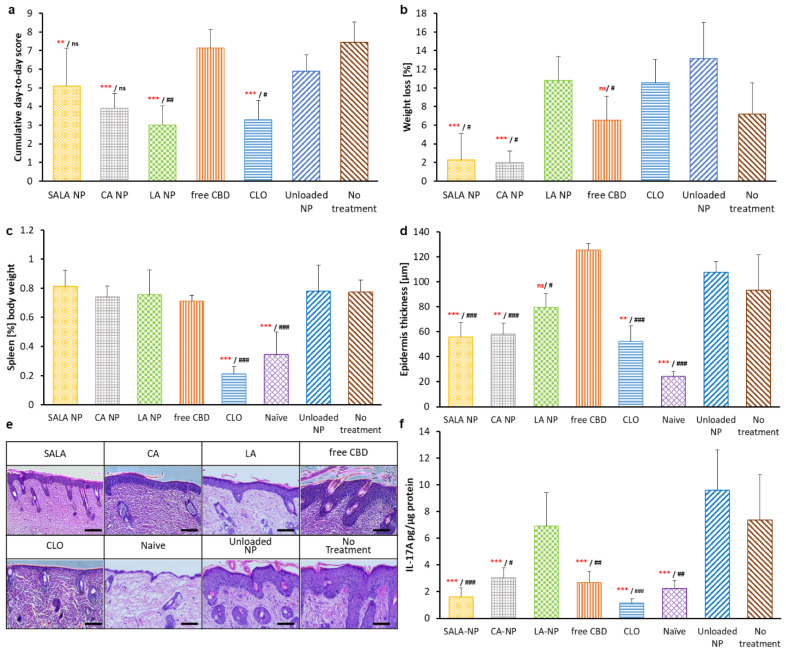
Comparison of the applied treatments with respect to the sum of their day-to-day cumulative PASI scores (**a**); Evaluation the mice body weight change from day 0 (D0) to day 5 (D5) (**b**); Evaluation of the spleen to body weight ratio [%] as an indication for systemic exposure of the treatments (**c**); Evaluation of the IMQ induced acanthosis measured as the epidermis thickness (**d**); Histological examination by H&E staining of the mice back skin samples (**e**); Evaluation of the IL-17A release in the mice back skin samples taken on D5 (**f**). ns/**/***—not significant/*p* < 0.01/*p* < 0.001 compared to the No treatment group, red symbols ns/#/##/###—not significant/*p* < 0.05/*p* < 0.01/*p* < 0.001 compared to the Unloaded NP treatment group, ANOVA followed by post-hoc Tukey test. Data are presented as mean ± SD. Scale bar: 100 µm.

To perform an overall comparison between the treatments throughout the study rather than on a day-to-day basis, we summed up the daily cumulative scores from day 0 to day 5 (Figure 5a). As shown in Figure 5a, All the G+ treatment groups were significantly more effective than the untreated group in reducing IMQ-induced psoriasis symptoms (*p* < 0.001 for CBD-loaded CA-NPs, CBD-loaded LA-NPs, and CLO, and *p* < 0.01 for CBD-loaded SALA-NPs, ANOVA). Unloaded LSNs were the most effective treatment in the G- group, being significantly less effective than treatments with CBD-loaded LA-NPs (*p* < 0.01) and CLO (*p* < 0.05). Among the G+ treatment groups, treatment with CBD-loaded SALA-NPs was the least effective, although no significant difference (*p* > 0.05) was noted compared to the other G+ groups. The differences between the LSNs stabilized with CA, LA, or SALA could be attributed to differences in the release rates of CBD from these NPs. NPs, such as those stabilized with LA, released CBD at a higher rate and, therefore, had a lower day-to-day score. In contrast, NPs stabilized with SALA released their CBD at a slower rate but allowed its accumulation, thus providing a similar effect on day 5.

#### 2.4.4. Weight Loss

Examination of body weight changes during the study and the spleen-to-body weight ratio did not show significant differences between the G+ and the G- groups (Figure 5b). Figure 5b shows that treatments with CBD-loaded SALA-NPs and CBD-loaded CA-NPs were the only treatments that significantly kept the body weight of the diseased mice almost constant compared to the untreated group. These treatments prevented the decrease in body weight, which was significantly different from all other treatments except for the f-CBD treatment group. Body weight is a common measure of an animal’s health condition, while its loss is usually associated with the IMQ model. Numerous studies have shown that the change in body weight in mice treated with topical imiquimod is related to reduced consumption of food and water [40,41]. Therefore, the extent of the weight loss reversal might be a measure of treatment effectiveness. Data available from previous studies regarding the influence of CBD on body weight have indicated that CBD induces weight loss by appetite depression through CB2 receptors in rats [42], as well as in humans [43]. Nevertheless, the effect of oral CBD on body weight in mice was significant only for the highest daily dose of 615 mg/kg/day, while no weight change occurred after lower doses of CBD [44]. For comparison, the total CBD dose applied on the psoriasis-like skin in the present study was about 50–70 mg/kg. It is interesting to note that topical application of clobetasol propionate used as a positive control in the IMQ-induced psoriasis model resulted in a marked decrease in body weight [45,46]. Thus, the significantly lower body weight loss in the groups treated with CBD-loaded SALA-NPs and CBD-loaded CA-NPs could be explained by their anti-inflammatory action that reversed the influence of IMQ.

#### 2.4.5. Spleen

The spleen is the second major immune organ besides the lymph nodes. The observed splenomegaly and the subsequent increase in spleen-to-body weight ratio indicates a systemic effect exerted by the treatment on the immune system. It is proposed that IMQ-related splenomegaly is caused by inflammation [47], whereas the hyposplenism induced by clobetasol (CLO) was associated with the depletion of splenic dendritic cells [48]. According to the observed results (Figure 5c), neither of the CBD-including treatments prevented IMQ-induced splenomegaly as CLO, possibly indicating localization of the NPs in the skin, preventing systemic exposure to CBD.

#### 2.4.6. Acanthosis Evaluation

The results obtained for the measured acanthosis values, as seen from Figure 5d,e, are well in line with the day-to-day cumulative scores, although without the distinct differences between the groups treated with the CBD-loaded LSNs. The most visually pronounced effect of CLO can be attributed to its anti-inflammatory activity as well as skin tissue atrophy generally associated with corticosteroid treatment [49].

#### 2.4.7. Anti-Inflammatory Action of CBD-Loaded LSNs as Evaluated by Reduction in IL-17A Secretion

Pro-inflammatory cytokines play a significant role in psoriasis disease manifestation. It was shown that the IMQ-induced psoriasis model is mediated via the IL-23/IL-17 axis [20]. Other publications showed the effectiveness of IL-17A antagonists and anti-IL-17A ssDNA aptamers in reversing the action of IMQ in mice [50,51]. Although CBD has not been tested previously in the IMQ-induced psoriasis model in mice, several other mice and human models have demonstrated its effectiveness in reducing IL-17A secretion [52,53]. In the present study, we have selected to evaluate the influence of various CBD-loaded LSNs on the secretion of IL-17A as a supplementary measure of their effectiveness.

The results showed that treatment with CBD-loaded SALA-NPs was more effective in the reduction in IL-17A secretion compared to treatment with CBD-loaded LA-NPs (*p* < 0.01, ANOVA) (Figure 5f). While CBD-loaded LA-NPs had no effect on IL-17A release, both treatments with CBD-loaded SALA-NPs and CBD-loaded CA-NPs resulted in a significant reduction in IL-17A levels compared to the negative control groups, the untreated group and the group treated with unloaded LSNs. The finding that treatment with CBD-loaded LA-NPs was less effective in inhibiting IL-17A compared to other CBD-loaded LSNs may be explained by an inherent pro-inflammatory activity of LA. Such activity was previously shown by the ability of LA to induce the release of IL-12 from the RAW264.7 cells (BALB/c mouse macrophages) [54] and the release of IP-10 chemokine from human U937 macrophages [55]. Considering IL-17A, LA was shown to promote differentiation of Th17 cells, resulting in increased levels of IL-17A [56]. The difference in the effects between CBD-loaded NPs containing LA and those containing SALA eutectic mixture can be attributed to the lower solubility and release of free LA molecules from the eutectic mixture. For example, the individual solubility of each lidocaine and prilocaine is significantly reduced when both components form a eutectic mixture [57]. A similar effect of toxicity reduction in eutectic mixture components was shown for the reduction in menthol toxicity on HaCaT cells when applied as a component of a eutectic mixture with either lauric, stearic, or myristic acids [58].

## 3. Materials and Methods

### 3.1. Nano-Particles Preparation

Ethyl cellulose (EC) up to about 0.05% *w*/*w*, one of the stabilizing lipids: CA or SALA (24:76)—0.025% *w*/*w*, or LA—0.05% *w*/*w*, Triethyl citrate (TEC)—0.05% *w*/*w*, and CBD—0.015% *w*/*w*, all were dissolved in absolute ethanol. The solution was constantly stirred on a magnetic plate at about 700 RPM. The ratio of magnetic stirrer length to beaker diameter was at least 1:3. Deionized water was added by dripping at a constant rate of about 22–25 mL/min with a syringe pump NE-300, New Era Pump Systems (Farmingdale, NY, USA) to the final content of about 60% *w*/*w* of the final dispersion mass. The obtained NP dispersion was evaporated with R-205 Rotavapor (Buchi Labortechnik AG, Flawil, Switzerland) until about four times volume reduction. To obtain concentrated NP dispersion, several consecutive centrifugation steps were performed. The CBD content in the obtained nanoparticles was determined by High Pressure Liquid Chromatography HPLC, and the entrapment efficiency percentage (EE%) was calculated according to the following Equation (1):

(1) $$\mathrm{EE}\%=\;\frac{\mathrm{Mass}\;\mathrm{of}\;\mathrm{CBD}\;\mathrm{in}\;\mathrm{formulation}}{\mathrm{Total}\;\mathrm{mass}\;\mathrm{of}\;\mathrm{CBD}\;\mathrm{used}\;\mathrm{for}\;\mathrm{formulation}}\times 100$$

### 3.2. Size and Microscopic Analysis

#### 3.2.1. Dynamic Light Scattering (DLS)

The hydrodynamic diameter spectrum of the NPs was collected using a CGS-3 Compact Goniometer System (ALV GmbH, Langen, Germany). The laser power was 20 mW at the HeNe laser line (632.8 nm). Correlograms were calculated by ALV/LSE 5003 correlator, which were collected at 90°, for 20 s for 10 times, at 25 °C. The NP size was calculated using the Stokes–Einstein relationship, and the analysis was based on the regularization method as described by Provencher [59].

#### 3.2.2. Nanoparticle Tracking Analysis (NTA)

Measurements were performed using a NanoSight NS300 instrument (Malvern Instruments Ltd., Worcestershire, UK), equipped with a 632 nm laser module and 450 nm long-pass filter, and a camera operating at 25 frames per second, capturing a video file of the particles moving under Brownian motion. The software for capturing and analyzing the data (NTA 3.4, Build 3.4.4) calculated the hydrodynamic diameters of the particles by using the Stokes–Einstein equation.

### 3.3. Determination of CBD in NP Dispersion

To quantify CBD (within nano-sized particles), to about 25 μL aliquots from each particle sample that was carefully weighed, 975 μL MeOH was added and stirred. After at least 10 min, the samples were further diluted 1:10, and then the liquid was injected into an HPLC system (Shimadzu VP series, Shimadzu Corp., Tokyo, Japan), equipped with a prepacked column (ReproSil-Pur 300 ODS-3, 5 µm, 250 mm 4.6 mm, Dr. Maisch, Ammerbuch, Germany), which was constantly maintained at 30 °C. The samples were chromatographed using a mobile phase of acetonitrile-35 mM acetic acid (75:25) at a 1 mL/min flow rate. A calibration curve, peak area measured at 208 nm versus CBD concentration, was constructed by running standard drug solutions in MeOH for each series of chromatographed samples.

### 3.4. In-Vitro Skin Penetration Study

#### 3.4.1. In-Vitro Skin Penetration

The penetration of CBD from CBD-loaded NP formulated in various gel formulations into the skin was determined in vitro using a Franz diffusion cell system (Permegear, Inc., Bethlehem, PA, USA). The diffusion area was 1.767 cm^2^ (15 mm diameter orifice), and the receptor compartment volume was 12 mL. The solutions in the water-jacketed cells were constantly set at 37 °C and stirred by externally driven, Teflon-coated magnetic bars. Each set of experiments was performed with twelve diffusion cells, each containing abdominal rat skin. The animal treatments were performed in accordance with a protocol reviewed and approved by the Institutional Committee for the Ethical Care and Use of Animals in Experiments, Ben-Gurion University of the Negev, which complies with the Israeli Law of Human Care and Use of Laboratory Animals”. Authorization number: IL-30-06-2020(C). Sprague–Dawley rats were euthanized by aspiration of CO_2_. Abdominal hair was carefully clipped, and sections of full-thickness skin were excised from the fresh carcasses of animals and used immediately. All skin sections were measured for transepidermal water loss (TEWL), and only those pieces in which TEWL levels were less than 10 g/m^2^/h were used. TEWL testing was performed on skin pieces using the Dermalab Cortex Technology instrument (Hadsund, Denmark). The skin was placed on the receiver chambers with the stratum corneum facing upwards, and the donor chambers were then clamped in place. The receiver chamber, defined as the side facing the dermis, was filled with phosphate buffer (pH 7.4)—ethanol 50:50 solution [60] to allow sink conditions. Formulated NPs (0.2 mL or approx. 200 mg) containing 100 μg (about 0.05% *w*/*w*) of entrapped CBD was applied on the skin at time = 0. After a 6-h experimental period, each exposed skin tissue was removed, washed with plenty of water, wiped carefully, and tape-stripped (×15) to remove CBD adsorbed in the stratum corneum. Penetrated levels in the skin tissues were determined after overnight methanol extraction by HPLC (see Section 3.3).

#### 3.4.2. Preparation of NPs for Formulation Carrier Selection and Optimization

The 16% *w*/*w* Poloxamer 407 gel NP dispersion was prepared by mixing previously prepared Poloxamer 407 (Kolliphor P 407™, BASF, Florham Park, NJ, USA) 20% *w*/*w* solution in deionized water stored at about 5 °C with NP dispersion and deionized water. The 7% MAS NP dispersion was prepared by mixing 10% *w*/*w* MAS (Veegum HV™, Vanderbilt, Norwalk, CT, USA) with NP dispersion and deionized water. The 3%, 4%, and 5% *w*/*w* colloidal silica gel NP dispersions were prepared by mixing previously prepared 10–11%*w*/*w* silica (CAB-O-SIL 530^®^, Cabot, Boston, MA, USA) gel with NP dispersion and deionized water. SALA-stabilized NPs were used for all comparative permeation studies intended for carrier selection and optimization.

### 3.5. In-Vivo Skin Penetration Study and Image Analysis

The in-vivo penetration study was performed on Sprague–Dawley rats. On the day prior to the experiment, the rats were anesthetized, and their abdominal hair was carefully clipped and depilated (Veet cream, Reckitt Benckiser, Chartres, France). On the day of the experiment, the rats were anesthetized, and Coumarin 6 (TCI, Tokyo, Japan) loaded NPs formulated in a 5% silica gel were applied at about 50 mg/cm^2^. After two hours, the rats were euthanized by aspiration of CO_2_. The abdominal skin was washed with plenty of water and removed from the carcasses. The animal treatments were performed in accordance with protocol authorization number: IL-30-06-2020(C). SALA stabilized, Coumarin 6 (C6) loaded NPs were prepared similarly to CBD loaded NPs (see Section 3.1), with initial 5 × 10^−4^% *w*/*w* C6 content in the ethanol solution. The C6-loaded NP dispersion in 5% silica gel was prepared as described in Section 3.4.2 with a final concentration of about 0.002%*w*/*w* C6. The excised skin was snap-frozen in liquid nitrogen and sectioned with a cryotome using 100µm thickness for further confocal microscopy analysis (Spinning disc confocal microscope, 3i, Denver, CO, USA). The micrographs were collected with 1.5 μm depth steps. Further, they were processed with Fiji software (version 2.9.0/1.54f) [61] using the Z-project function.

### 3.6. Imiquimod-Induced Psoriasis in Mice

#### 3.6.1. Animals

For the study, male C57BL/6 8 to 11-weeks-old mice were used. The animals were kept under standard conditions with free access to water and food. The animal treatments were performed in accordance with a protocol reviewed and approved by the Institutional Committee for the Ethical Care and Use of Animals in Experiments, Ben–Gurion University of the Negev, which complies with the Israeli Law of Human Care and Use of Laboratory Animals”. Authorization number: IL-64-11-2021(C).

#### 3.6.2. Preparation of the Formulated NP Dispersions and the CBD Emulsion

The formulated NP dispersions were prepared by mixing concentrated dispersions of CBD-loaded NP stabilized with either CA, LA, SALA or unloaded NP with 10% *w*/*w* silica gel (CAB-O-SIL 530^®^, see Section 3.4.2) aiming at a final concentration of 5% *w*/*w* silica. Since the lowest achieved CBD content for these dispersions was 1.25% *w*/*w* (12.5 mg/g for CA NP see Table 1), the content of CBD in the formulated dispersions was set to 0.6% *w*/*w*. The content of unloaded NP was determined by the highest achievable NP concentration, allowing to obtain 5% *w*/*w* silica gel resulting in about 8 × 10^12^ NP/g (based on initial 1.6 × 10^13^ NP/mL for Unloaded NP—see Table 1).

CBD emulsion was prepared by dissolving CBD (4.6% *w*/*w*) in C8-C10 triglycerides (Miglyol 810, Cremer Oleo, Hamburg, Germany) and emulsifying with Polysorbate-80 (J.T. Baker, Phillipsburg, NJ, USA) and deionized water. The prepared emulsion was mixed and dispersed with about 10% silica gel (CAB-O-SIL 530^®^, see Section 3.4.2) to obtain the following final contents (*w*/*w*): CBD—0.6%, Miglyol 810—13%, Polysorbate-80—2%, silica gel—5%, and deionized water ad 100%.

#### 3.6.3. Imiquimod-Induced Psoriasis in Mice

**Scheme 1:**
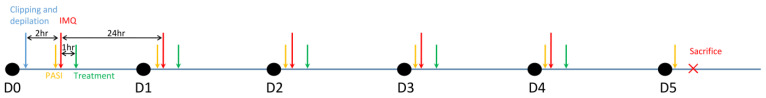
IMQ-induced psoriasis in mice conduct.

On the first study day (D0), the back of the mice was shaved using an electric clipper and depilated (Veet cream, Reckitt Benckiser, Chartres, France), see Scheme 1. The mice with large black pigmentation areas on their skin—anagen areas, resulting in quick hair regrowth [62] and preventing the effective application of treatments, were excluded from the study. These animals were sacrificed on the first study day, n = 5. Samples obtained from these mice were designated “naive” and served as an IMQ non-treated control. Other experimental groups received a daily topical dose of 62.5 mg of commercially available IMQ cream (5%) (Aldara, 3M, Bracknell, UK) on the back for 5 consecutive days to establish a model of IMQ-induced psoriasis [20]. Negative control (No Treatment—NT) mice did not receive additional treatment on top of IMQ. Due to a large number of treatments, the experiments were performed in two series, each one including the NT group (n = 6 and n = 5) as an internal control. The presented data is for the combined result of both NT groups (n = 11). For other groups, the treatment was applied 1 h after the application of IMQ cream. The CA (n = 5), LA (n = 6), SALA (n = 7), and Unloaded (n = 5) groups received a daily dose of 50 mg of formulated CA, LA, and SALA-stabilized CBD-loaded NPs and formulated LA stabilized unloaded NPs, respectively. The Blank group served as a negative control for formulated CBD-loaded NPs. The formulated NPs’ dispersions were prepared in 5% silica gel, as described in Section 3.6.2. The free CBD (f-CBD) group received a daily dose of 50 mg of formulated CBD emulsion (see Section 3.6.2). The CLO group received a daily dose of 100 mg 0.05% commercial clobetasol cream (Clobetasol 0.05%, Trima, Maabarot, Israel), serving as a positive control.

#### 3.6.4. Evaluation of Psoriasis Area Severity Index (PASI) Score and Spleen Index

PASI was used to assess the inflammatory condition in the mice on all study days (D0-D5). For this purpose, we visually examined each mouse’s back skin erythema (redness) and scaling. The skin thickness was measured by a calibrated caliper (Mitutoyo, Japan) as the thickness of a back skin fold. The visually examined parameters were assigned a score between 0 and 4 (0-none, 1-slight, 2-moderate, 3-severe, 4-very severe) [63]. To accommodate the possible differences in evaluation between the series of experiments, the values obtained for each mouse were normalized by the maximum value obtained for all mice and multiplied by 4. To compare with the visually examined parameters, the skin thickness score was calculated as the thickness change from D0 for each mouse normalized by the maximum thickness change for all mice and then multiplied by 4 to bring the value to the 0–4 scale. The cumulative score (0 to 4), calculated as the average of the erythema, scaling, and thickness scores, indicates the severity of psoriasis inflammation (PASI score).

The animal weight was measured on the study’s first (D0) and last (D5) days. Following the sacrifice, the spleens were weighed, and the spleen index was calculated as the percent of the animal weight on D5.

#### 3.6.5. Histology

For histological analysis, following the sacrifice, the back skin samples were removed and fixed with 10% formaldehyde, embedded into paraffin blocks, cut, and finally stained with hematoxylin-eosin (H&E). The tissue sections on slides were then micrographed with a light microscope (Nikon Eclipse Ts2, Tokyo, Japan) with ×20 magnification. For each slide (corresponding to a specific animal), 12 micrographs were taken. The epidermal thickness was evaluated through area and length measurements [50] using Fiji software (version 2.9.0/1.54f) [61]. To avoid bias, the area of the viable epidermis containing the blue-purple stained nuclei was measured with the “wand” function. The epidermal thickness for each mouse was calculated as an average of 12 measurements.

#### 3.6.6. Evaluation of IL-17A Levels in the Skin Tissue

For IL-17A evaluation, following the sacrifice, the back skin samples were snap-frozen and then dehydrated in a lyophilizer. The lyophilized samples were ground in a mortar with column sand (Sigma-Aldrich Inc., St. Louis, MO, USA) and then homogenized in a Tissue Extraction Reagent I (Thermo Fischer Scientific, Waltham, MA, USA) with Polytron homogenizer (Kinematica, Malters, Switzerland). Homogenates were centrifuged for 5 min at 10,000 RPM, and the supernatants were analyzed for protein and IL-17A content. The protein content was analyzed with Bradford assay using Protein Assay Dye Reagent Concentrate (Bio-Rad Laboratories, Hercules, CA, USA). The IL-17A content was evaluated by enzyme-linked immunosorbent assay (ELISA). The ELISA was performed according to the manufacturer’s instructions for the kit (ELISA Max, Biolegend, San Diego, CA, USA). The results for each skin extract (corresponding to a specific animal) were obtained by normalization of the IL-17A content by the protein content of each sample.

### 3.7. Statistical Analysis

Data analysis was performed using the Graph-Pad Prism software (Version 5.01, San Diego, CA, USA). Data were expressed as mean ± standard deviation (SD) or original data represented. The one-way analysis of variance (ANOVA) followed by Tukey’s post hoc test was used to compare groups. The *p*-value < 0.05 was considered statistically significant.

## 4. Conclusions

The present study demonstrates the potential of LSNs as a versatile platform for precisely delivering CBD to the skin. Formulation studies involving fumed silica, MAS, and Poloxamer thermogel underscored the critical role of vehicle selection and optimization for effective nanoparticle dispersion. In vitro skin permeation testing revealed that fumed silica was significantly more effective than other gelling agents. The in-vivo assessments using LSNs loaded with fluorescent marker C6 confirmed successful permeation and localization within the viable epidermis. Utilizing an IMQ-induced psoriasis model in mice provided additional evidence of CBD’s efficacy in treating psoriasis. CBD-loaded LSNs significantly reduced the PASI score and acanthosis, as well as inhibited the IL-17A release compared to the control treatment groups, indicating a substantial improvement in psoriasis symptoms. Moreover, the difference in response between CBD-loaded LSNs and CBD emulsion suggests deeper skin penetration and localization due to the LSN formulation. Lastly, the variation in the anti-inflammatory response among LSNs stabilized with CA, LA, or SALA highlighted the significance of the stabilizing lipid selection, as LSNs stabilized with CA and SALA demonstrated greater anti-inflammatory effect compared to LA-stabilized LSNs. This has emphasized the importance of lipid selection in topical drug delivery.

## Figures and Tables

**Table 1:** Nanoparticles and their parameters.

**LSN-Type**	**Size [nm]**	**PDI**	**NP Conc. [NPs/mL]**	**CBD Conc. [mg/g]**	**EE [%]**
SALA	238.0	0.028	2.06 × 10^13^	13.8	86.3
CA	220.8	0.102	1.52 × 10^13^	12.5	69.2
LA	245.0	0.182	1.78 × 10^13^	20.0	68.0
Unloaded	226.2	0.114	1.60 × 10^13^	NA	NA

## Data Availability

Data is available upon request.
